# Meniscectomy is still a frequent orthopedic procedure: a pending need for education on the meniscus treatment possibilities

**DOI:** 10.1007/s00167-021-06612-w

**Published:** 2021-06-04

**Authors:** Paweł Bąkowski, Kamilla Bąkowska-Żywicka, Kinga Ciemniewska-Gorzela, Tomasz Piontek

**Affiliations:** 1 Department of Orthopedic Surgery, Rehasport Clinic, Górecka Street 30, 60-201 Poznan, Poland; 2 Institute of Bioorganic Chemistry Polish Academy of Sciences, Noskowskiego 12/14, 61-704 Poznan, Poland; 3 Department of Spine Disorders and Pediatric Orthopedics, University of Medical Sciences, Poznan, Poland

**Keywords:** Knee arthroscopy, Meniscus repair, Survey, Surgical expertise

## Abstract

**Purpose:**

The purpose of this study was to evaluate the current status of education of polish surgeons in the subject of meniscus repair possibilities. The analysis of the possible correlations between the number of knee arthroscopy procedures performed by polish surgeons and their decision whether to remove or to repair the damaged meniscus has been performed.

**Methods:**

Two-hundred and five registered orthopedic surgeons took part in surveys. The questionnaire contained the description of 20 patients with different types of meniscus damage and three questions concerning the experience in knee arthroscopy (two questions) and a choice of the treatment method (one question). Comparisons were made between knee arthroscopy experts (> 100 arthroscopies performed per year) and non-experts (≤ 100 cases).

**Results:**

The questionnaire was completed by 194 knee surgeons from Poland with different levels in knee arthroscopy experience. For most cases, experts and non-experts agreed on the meniscus treatment method. Statistically significant differences in the recommended treatment between experts and non-experts were observed in 4 cases, where experts decided to repair the damage rather than to perform the meniscectomy.

**Conclusions:**

Meniscectomy remains a frequent orthopedic procedure, despite meniscal sparing having been advocated for several decades now and despite the existence of meniscus repair technique which gives good clinical outcomes—augmentation of the damaged meniscus with a collagen membrane. Polish surgeons still need education on the meniscus treatment possibilities.

**Level of evidence:**

V.

## Introduction

Meniscal tears may be arthroscopically treated with resection, repair or replacement of the damaged tissue. In 2016 European Society for Sports Traumatology, Knee Surgery and Arthroscopy (ESSKA) has released the consensus guidelines to help the surgeon in the decision process [3]. The main finding was that the meniscectomy should not be proposed as a first-line treatment. The second European Consensus has studied the epidemiology, diagnosis and treatment of traumatic meniscal tears [16]. It follows the first consensus on the management of degenerative meniscal lesions.

The era of meniscal preservation is based on three pillars: (i) repair of the torn meniscus whenever reasonable, (ii) non-surgical treatment of asymptomatic meniscal pathologies despite a meniscal tear according to MRI, (iii) partial meniscectomy and resection of as much as necessary and as little as possible [5]. In this regard, the most amenable tears to be repaired are acute, traumatic tears within the peripheral well-vascularized red-red zone which are longitudinal-vertical in orientation [22]. In traumatic tears, the first choice is a repair or non-removal [4, 12]. The extended indications for the meniscal repair have been recommended for the subsequent clinical entities: horizontal cleavage tears in young athletes, root tears, ramp lesions, radial tears and tears in the red-white zone [7–10, 13, 17, 19–21].

A large-scale national study has clearly shown that the number of meniscus repairs is increasing, however, the meniscectomy is still frequent worldwide [1, 14, 15, 23], despite a growing evidence of very good results of meniscus repair techniques. Selecting the most appropriate treatment for a given patient involves both patient factors (e.g., age) and tear characteristics (e.g., location of tear). It is, therefore, reasoned that a national study is needed in Poland. Therefore, the purpose of the study was to determine the current status of education of polish surgeons in the subject of meniscus repair possibilities via the analysis of the trends in meniscus tear treatment (meniscal resection versus meniscal repair) in the environment of the Polish orthopedists. The hypothesis was that Polish surgeons have been following the worldwide trends in meniscal resection and meniscal repair and possibly have been using the meniscectomy even in cases that are considered repairable.

## Material and methods

### Cases

**Fig. 1:**
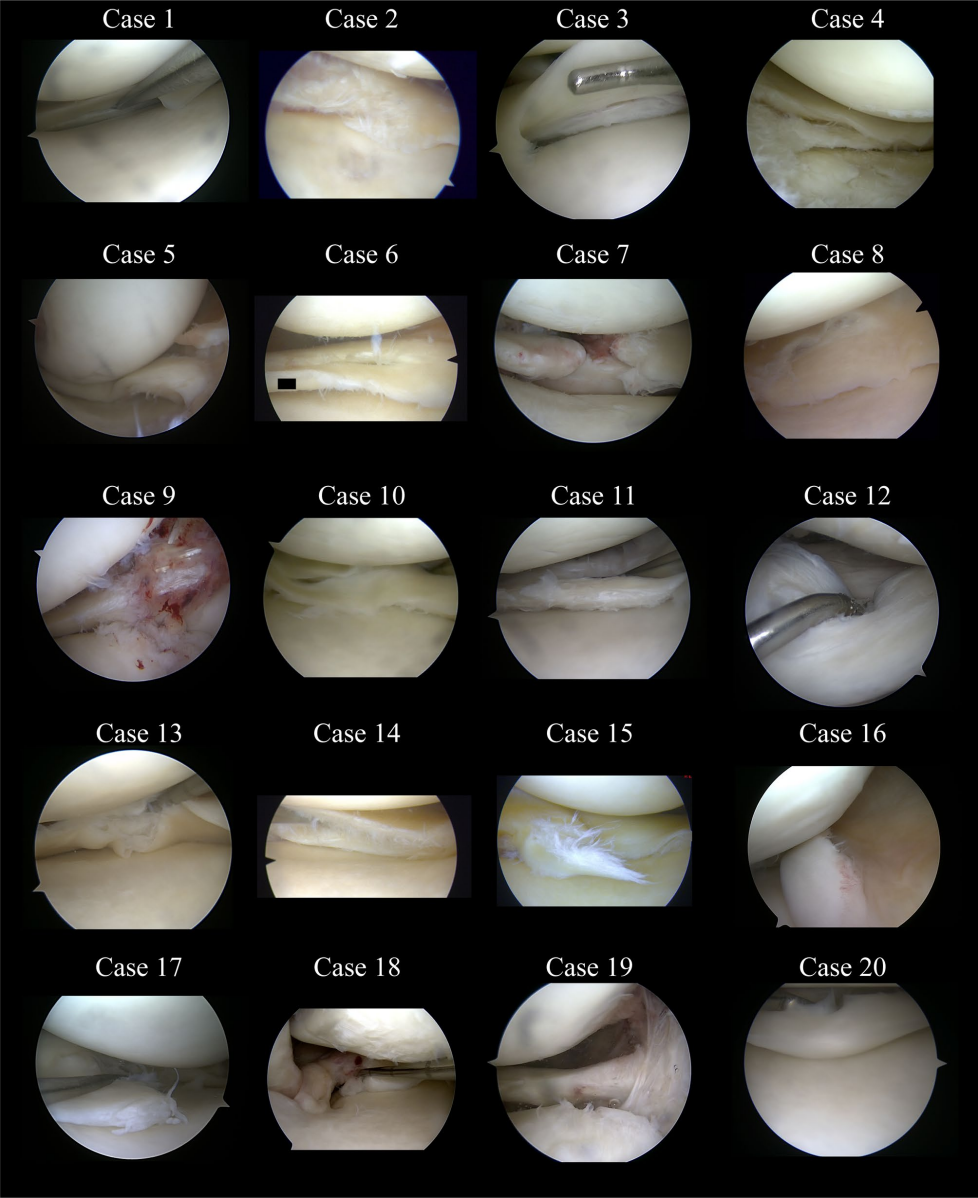
Arthroscopic images of the meniscus in 20 studied cases

For this study 20 case reports with the meniscal injuries that differed in morphology, patient age, chronicity and the method of a prior treatment (Table 1 and Fig. 1) have been chosen. Based on authors’ expertise and worldwide trends and recommendations, these cases have been classified into three groups:

- *Group I*—cases that are considered repairable (cases: 1, 3, 5, 7, 9, 12, 16, 18, 19, 20),
- *Group II*—cases considered unrepairable (cases 11 and 17),
- *Group III*—cases requiring biological support, e.g. augmentation with collagen membrane (cases 2, 4, 6, 8, 10, 13, 14, 15).

**Table 1:** Characteristics of 20 cases presented to the orthopedists in the survey

No.	Sex	Age	Damage localization	Damage description
1	Male	34 y.o.	Medial meniscus	Longitudinal tear
2	Male	34 y.o.	Medial meniscus	Complex tear
3	Male	18 y.o.	Lateral meniscus body	Longitudinal tear
4	Male	39 y.o.	Lateral meniscus body	Complex tear
5	Male	33 y.o.	Medial meniscus	Bucket handle tear
6	Male	23 y.o.	Medial meniscus	Longitudinal tear, previously treated with suture
7	Female	40 y.o.	Lateral meniscus body	Radial tear
8	Male	47 y.o.	Medial meniscus body	Radial tear
9	Female	38 y.o.	Lateral meniscus	Posterior root
10	Female	51 y.o.	Medial meniscus	Complex tear
11	Female	20 y.o.	Medial meniscus	Complex tear
12	Male	23 y.o.	Medial meniscus	Longitudinal tear
13	Female	44 y.o.	Medial meniscus	Horizontal tear
14	Female	28 y.o.	Medial meniscus	Horizontal tear
15	Male	40 y.o.	Medial meniscus	Complex tear
16	Male	17 y.o.	Medial meniscus	Bucket handle tear, previously treated with suture
17	Male	30 y.o.	Medial meniscus	Complex tear
18	Female	63 y.o.	Medial meniscus	Posterior root
19	Male	65 y.o.	LATERAL meniscus	Posterior root
20	Male	35 y.o.	medial meniscus	Bucket handle tear

The treatments for all patients were performed by 2 orthopedists, PB and TP. The authors possess the knowledge about postoperative fate of the presented cases.

### Study design

A questionnaire has been presented to 205 orthopedists with various levels of clinical expertise in arthroscopy during Polish Arthroscopy Society Congress (24–26 October 2019, Katowice, Poland). Participants have been questioned by 5 hostesses. The same cohort of orthopedists took part in our previous research [2]. Only fully completed survey forms (194) have been used for the analysis.

The questionnaire contained the description of 20 cases with different types of meniscus damage (Table 1 and Fig. 1) and 3 questions:

- *Question 1* How many arthroscopies do you perform per year?
  Possible answers: 0–10, 10–30, 31–50, 50–100, 100–200, 200–500, >500.
- *Question 2* How many arthroscopies have you performed in your carrier?
  Possible answers: 0–10, 10–30, 31–100, 100–500, 500–1000, 1000–2000, >2000.
- *Question 3* What type of meniscus treatment would you suggest for each patient?
  Possible answers: remove, repair.

The expert surgeons have been defined as surgeons performing more than 100 knee arthroscopies per year independently. The non-expert surgeons have been surgeons performing up to 100 knee arthroscopies per year. This should provide information on the treatment habits of the knee arthroscopy experts and non-experts and show whether the level of knowledge in terms of the meniscus damage diagnostics and treatment is linked to the level of experience. Of the surgeons, 55 (26%) have been classified as “experts”, whereas the remaining 144 (74%) participants have been defined as “non-experts”.

### Statistical analysis

The statistics have been conducted using Prism8 software (GraphPad Software, San Diego, CA). The power analysis has been conducted to identify the minimum number of the participants required in each group to detect the statistical significance. The sample size calculation showed that with a power of 80% (2-sided testing at a significance level of 0.05), a cohort size of 43 participants was needed. To test proportional differences in categorical variables, a Chi-square test was performed. Fisher exact tests were used when cells contained less than five subjects. Statistical significance was determined as *p* < 0.05.

## Results

### Cases

Most of the surgeons would perform meniscus repair in 50% of cases: 1, 3, 5, 8, 9, 12, 14, 18, 19 and 20. The meniscus removal would be performed by surgeons in cases: 2, 4, 6, 7, 10, 11, 13, 15, 16 and 17.

### Experts versus non-experts

This chapter compares the responses received from expert and non-expert surgeons. Experts were choosing an option “repair” 539 times (54% of all experts’ answers) and non-experts—1 394 times (48%, n.s.). The results are presented in Table 2. For most cases, experts and non-experts agreed on the meniscus treatment method. Statistically significant differences in the recommended treatment between experts and non-experts were observed in 4 cases, where experts decided to repair the damage more frequently: case 5 (33 y.o. male, bucket handle tear in medial meniscus), case 9 (38 y.o. female, posterior root in lateral meniscus), case 18 (63 y.o. female, posterior root in medial meniscus) and case 19 (65 y.o. male, posterior root in lateral meniscus).

**Table 2:** Comparison of the results of the survey between experts and non-experts

Case	Decision	Experts (*n* = 50)	Non-experts (*n* = 144)	*p* value
Cases considered repairable				
1	Remove Repair	2 (3%) 53 (97%)	15 (10%) 135 (90%)	n.s.
3	Remove Repair	5 (9%) 50 (91%)	8 (5%) 142 (95%)	n.s.
5	Remove Repair	6 (11%) 49 (89%)	51 (34%) 149 (66%)	0.002
7	Remove Repair	46 (84%) 9 (16%)	121 (81%) 29 (19%)	n.s.
9	Remove Repair	4 (7%) 51 (93%)	48 (32%) 102 (68%)	< 0.001
12	Remove Repair	5 (9%) 50 (91%)	27 (18%) 123 (82%)	n.s.
16	Remove Repair	27 (49%) 28 (51%)	91 (57%) 64 (43%)	n.s.
18	Remove Repair	15 (27%) 40 (73%)	69 (46%) 86 (54%)	0.047
20	Remove Repair	5 (9%) 50 (91%)	19 (13%) 131 (87%)	n.s.
Cases considered unrepairable				
11	Remove Repair	44 (80%) 11 (20%)	126 (84%) 24 (16%)	n.s.
17	Remove Repair	51 (93%) 4 (7%)	123 (82%) 27 (18%)	n.s.
Cases requiring biological support				
2	Remove Repair	41 (78%) 12 (22%)	111 (74%) 39 (26%)	n.s.
4	Remove Repair	39 (71%) 16 (29%)	100 (67%) 50 (33%)	n.s.
6	Remove Repair	36 (65%) 19 (35%)	89 (59%) 61 (41%)	n.s.
8	Remove Repair	30 (54%) 25 (46%)	72 (48%) 78 (52%)	n.s.
10	Remove Repair	49 (89%) 6 (11%)	119 (79%) 31 (21%)	n.s.
13	Remove Repair	36 (75%) 14 (25%)	100 (67%) 50 (33%)	n.s.
14	Remove Repair	17 (31%) 38 (69%)	55 (37%) 95 (63%)	n.s.
15	Remove Repair	49 (89%) 6 (11%)	127 (85%) 23 (15%)	n.s.

*n.s.* not significant

*p* value is presented to establish statistical significance between expert and non-expert treatment

For most cases, no correlation between the number of knee procedures performed per year and the decision to repair or to remove the meniscus was observed. The only differences were noticed for: case 4 (most surgeons who performed 31–50 knee arthroscopies per year recommended meniscus repair), case 5 (most surgeons who performed 51–100 knee arthroscopies per year recommended meniscus removal), case 9 (the least experienced surgeons, who performed up to 10 knee arthroscopies per year, recommended meniscus removal) and case 16 (most surgeons who performed up to 30 and 51–100 knee arthroscopies per year recommended meniscus removal). In case 2 and case 13 the most experienced surgeons, who performed more than 500 knee arthroscopies per year recommended meniscus repair.

## Discussion

The most important finding of the present study was that meniscectomy remains a frequent orthopedic procedure, despite meniscal sparing having been advocated for several decades now and despite the existence of meniscus repair technique which gives good clinical outcomes—augmentation of the damaged meniscus with a collagen membrane [6, 11, 18]. Polish surgeons still need education how to save the damaged meniscus.

The agreement between expert and non-expert arthroscopic knee surgeons in the decisioning whether to remove or to repair the damaged meniscus has been observed. 50% of cases were qualified by all questioned polish surgeons (experts and non-experts) for meniscal resection and 50% for meniscal repair.

There is a general agreement about preserving the meniscus whenever possible [12]. Despite that, in 50% (10 out of 20) cases: 2, 4, 6, 7, 8, 10, 11, 13, 15, 17 a consensus has been observed among young surgeons and expert surgeons to remove the damaged menisci. These are the examples of longitudinal tear (6), complex tears (2, 4, 10, 11, 15, 17), radial tears (7, 8) and horizontal tear (13). In the past, the principles of arthroscopic surgery recommended resecting meniscal tissue until a stable peripheral rim was obtained and obviously polish surgeons followed these recommendations in these 10 cases. However, in this study we have hypothesized, that polish surgeons would classify only cases 11 and 17 as non-repairable and the rest (2, 4, 6, 8, 10, 13, 14 and 15)—as cases requiring biological support, e.g. augmentation with collagen membranes. In this latter group, there are patients from our recent study [18]. It has been demonstrated that even patients with combined (horizontal and radial or longitudinal component) and complex meniscal tears (tear extended through avascular zones or/and composed with two or more morphological tear pattern) could be treated with an "all-inside" arthroscopic suture of meniscus and augmentation with a collagen membrane technique with bone marrow blood injection. with good or very good All treated patients had a good or very good clinical outcomes after 2 [18] and 5 years [11] and they did not require reoperation. Augmentation with a collagen membrane method has been already published by us and presented on several well-recognized international conferences, therefore it is difficult to explain the fact that the infestation is rare due to the lack of access to information and education on new methods of meniscus repair. One would rather suspect that not common use of meniscus augmentation with a collagen membrane is caused by the costs of the procedure—lack of reimbursement by Polish National Health Fund and the need of special training.

In this study, a consensus has been reached between young and expert surgeons to repair the meniscus in 30% (6 of 20) studied cases: cases 1, 3, 12, 14, 16, 20. However, it has been hypothesized that the repair group would comprise also cases 5, 7, 9, 12, 18 and 19. Case 5 is a bucket handle damage, that is 89% repairable by experts, as opposed to non-experts (66%). Additionally, the experts would prefer to repair meniscal root tears (cases 9, 18, 19), which implicates that they are aware that the treatment of the root tears is of great importance in the prevention of knee arthrosis. However, there was a greater reluctance to repair the posterior root tear noticed by non-experts in older patients (case 18 and 19–63 and 65 years old, respectively). One could imagine that younger surgeons are not yet well trained in meniscus repair methods. There is a need for training, education and increasing awareness, especially in young surgeons.

More than half of questioned surgeons (both experts and non-experts) decided to recommend the remove a recurrent bucket handle tears (cases 6 and 16). This is a poor results, taking into considerations patients age (17 and 23 y.o.) and worldwide recommendations. Possibly the decision to remove might be dictated by a reluctance to take responsibility for a possible subsequent unsuccessful treatment.

This study had limitations. Although 100% of the participants returned the questionnaires, some of them were not fully completed and therefore excluded from the final analysis which reduced the sample size. Bias may have been introduced due to the invitation of surgeons taking part in the Polish Arthroscopy Society Congress, which also limited the cohort size. Defining the level of expertise at a cutoff of 100 knee arthroscopies per year independently could be considered a biased decision for this study.

The clinical relevance of the work is important. Meniscus resection is still widely used in Poland and Polish surgeons need a proper education on the meniscus treatment possibilities.

## Conclusions

Polish surgeons follow the worldwide trends in the management of acute and chronic meniscal tears. Meniscectomy still remains a frequent orthopedic procedure, despite meniscal sparing having been advocated for several decades now. There is a disproportion between the conclusions drawn from scientific studies in favor of conservation and actual practice. Meniscus resection is still widely used in Poland, despite the existence of meniscus repair technique which gives good clinical outcomes—augmentation of the damaged meniscus with a collagen membrane. Polish surgeons still need education on the meniscus treatment possibilities.
